# Cache-Oblivious parallel SIMD Viterbi decoding for sequence search in HMMER

**DOI:** 10.1186/1471-2105-15-165

**Published:** 2014-05-30

**Authors:** Miguel Ferreira, Nuno Roma, Luis MS Russo

**Affiliations:** 1 Instituto Superior Técnico, Universidade de Lisboa, Av. Rovisco Pais, 1049-001 Lisboa, Portugal; 2 INESC-ID, Rua Alves Redol, 9, 1000-029 Lisboa, Portugal

**Keywords:** Sequences alignment, Hidden Markov model, Viterbi, HMMER, Parallelization, Streaming SIMD Extensions (SSE)

## Abstract

**Background:**

HMMER is a commonly used bioinformatics tool based on Hidden Markov Models (HMMs) to analyze and process biological sequences. One of its main homology engines is based on the Viterbi decoding algorithm, which was already highly parallelized and optimized using Farrar’s striped processing pattern with Intel SSE2 instruction set extension.

**Results:**

A new SIMD vectorization of the Viterbi decoding algorithm is proposed, based on an SSE2 inter-task parallelization approach similar to the DNA alignment algorithm proposed by Rognes. Besides this alternative vectorization scheme, the proposed implementation also introduces a new partitioning of the Markov model that allows a significantly more efficient exploitation of the cache locality. Such optimization, together with an improved loading of the emission scores, allows the achievement of a constant processing throughput, regardless of the innermost-cache size and of the dimension of the considered model.

**Conclusions:**

The proposed optimized vectorization of the Viterbi decoding algorithm was extensively evaluated and compared with the HMMER3 decoder to process DNA and protein datasets, proving to be a rather competitive alternative implementation. Being always faster than the already highly optimized ViterbiFilter implementation of HMMER3, the proposed Cache-Oblivious Parallel SIMD Viterbi (COPS) implementation provides a constant throughput and offers a processing speedup as high as two times faster, depending on the model’s size.

## Background

### Sequence alignment algorithms

One of the most used alignment algorithms for sequence homology search is the Smith-Waterman algorithm [1]. It computes the optimal local alignment and the respective similarity score between the most conserved regions of two sequences, with a complexity proportional to $O(N^{2})$. The algorithm is based on a Dynamic Programming (DP) approach that considers three possible mismatches: insertions, deletions, and substitutions. To ensure that a local alignment is found, the computed scores are constrained to a minimum value of 0, corresponding to a restart in the alignment. To circumvent the computational complexity of the Smith-Waterman and similar alignment algorithms, alternative heuristic methods (like BLAST [2]) were developed. However, their lower complexity is obtained at the cost of sacrificing the resulting sensibility and accuracy.

**Figure 1:**
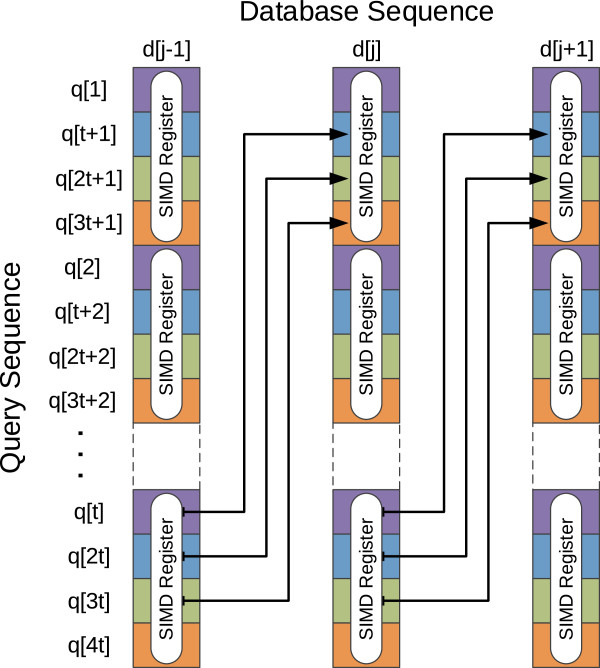
**Interleaved decomposition pattern proposed by Farrar **[3]**.**

An effective way that has been adopted to speed up these DP alignment algorithms is the exploitation of data-level parallelism. One of the most successful parallelization methods was proposed by Farrar [3], who exploited vector processing techniques using the Intel SSE2 instruction set extension to implement an innovative striped data decomposition scheme (see Figure 1). In his approach, each vector contains several cells from the same column of the scoring matrix. However, contrasting to other implementations, these cells are not contiguous. Instead, they are exactly *K* cells apart, in order to minimize the inter-row dependencies. Essentially, this processing pattern assumes that there is no dependencies across the vertical ‘segment sections’ (continuous sections). Whenever this assumption is not verified, the existing data dependencies have to be solved by a second inner loop (the *Lazy-F loop*). Since these vertical dependencies among cells are unlikely (although still possible), the resulting algorithm proves to be very effective in the average case.

**Figure 2:**
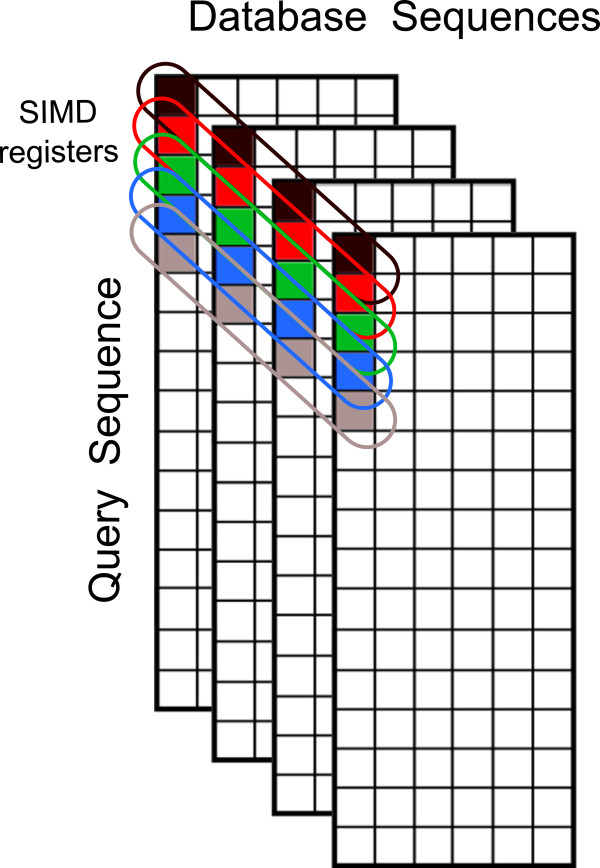
**Decomposition pattern proposed by Rognes in SWIPE **[4]**.**

Meanwhile, Rognes proposed a different method in his Swipe tool [4], which achieved even better performances than Farrar’s. Contrasting to Farrar’s, which was based on the exploitation of *intra*-task parallelism, Rognes’ method also makes use of SSE2 vector processing but exploits an *inter*-task parallelism scheme (i.e., multiple alignment tasks are run in parallel), by using a lock-step processing model (see Figure 2). Each vector is loaded with *N* different sequences, one in each vector element (or channel), and the algorithm concurrently aligns them against a target sequence, by using the *N* vector channels to hold the independent computed values. The drawbacks of this strategy are concerned with its restrictive application domain, resulting from the fact that the *N* alignments proceed coalesced, from the beginning to the end. Any divergence on the program flow carries a high performance penalty, either as stoppage time or as wasted computing potential (e.g., empty padded cells). Even so, the complete elimination of data dependencies between the values inside the same SSE register allows this technique to achieve *quasi*-optimal speed-ups. Therefore, this software implementation is often regarded as the fastest choice.

Other authors have even focused on the use of more specialized hardware architectures, such as GPUs [5], ASICs [6], or on parallelizing the algorithm onto a multi-node grid, usually by dividing the sequence database in blocks and independently searching on each block.

### Markov models and Viterbi decoding

Instead of searching with a single query sequence, several applications have adopted a previously built consensus, conveniently defined from a family of similar sequences. This consensus structure is usually known as a *consensus profile* and it provides a more flexible way to identify homologs of a certain family, by highlighting the family’s common features and by downplaying the divergences between the family’s sequences.

A common method to perform a profile homology search rests on a well-known machine learning technique: Hidden Markov Modelss (HMMs). As an example, an HMM may be constructed to model the probabilistic structure of a group of sequences, such as a family of proteins. Such resulting HMM is then used to search within a sequence database, by computing the probability of that sequence being generated by the model. HMMs may also be used to find distant homologs, by iteratively building and refining a model that describes them (such as in the SAM tool [7]).

**Figure 3:**
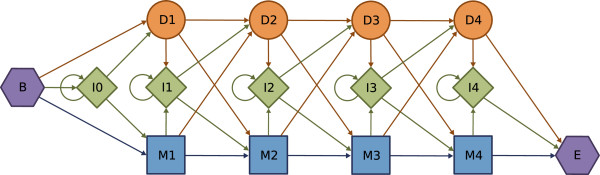
HMM for global alignment.

In 1994, Krogh *et al*. [8] developed a straightforward and generalized profile HMM for homology searches that emulates the results of an optimal alignment algorithm. The model is mainly composed by three different types of states, corresponding to matches/mismatches (M), insertions (I) and deletions (D), with explicit transitions between the three types of states. Figure 3 depicts an example of such model, where the match states (M) are represented by squares, the insertions (I) by rhombus and the deletions (D) by circles. The model also contains an initial and a final state, represented by hexagons.

The most important algorithms to process HMMs are the *Forward* algorithm, which gives the full probability for all possible model state paths; and the *Viterbi*’s algorithm, used to compute the most likely sequence of model states for the generation of the considered sequence. The complete path of states that is extracted by the application of Viterbi’s procedure thus corresponds to an optimal alignment of the considered sequence against the profiled model.

Hence, for a general Markov model, Viterbi’s algorithm computes the most likely sequence of hidden states. By denoting as *P*(*V*_*j*_(*i*)) the probability that the most likely path at time *i* ends at *V*_*j*_, Viterbi’s algorithm defines the following relation to compute this probability:

(1) $$\begin{array}{c} P(V_{j}(i))=P(x_{i}|V_{j})\underset{j^{\prime }}{\max}\{V_{j^{\prime }}t_{j^{\prime }j}\} \end{array}$$

In this equation, *P*(*x*_*i*_|*V*_*j*_) represents the probability of observing *x*_*i*_ in state *V*_*j*_. The $t_{j^{\prime }j}$ term represents the transition probability from state $V_{j^{\prime }}$ to state *V*_*j*_.

These equations are very similar to the corresponding recurrences of the Forward algorithm, with Viterbi’s using a *maximum* operation while Forward uses a *sum*. To avoid possible underflows resulting from the repeated products, the involved computations usually use logarithmic scores (*log-odds*). This conversion also replaces the multiplication operations by sums, which further simplifies the calculations. To simplify this *log-odds* notation, the term *V*_*j*_(*i*) will herein represent log(*P*(*V*_*j*_(*i*))). The recurrence equations of Viterbi’s algorithm, for the profile HMMs, in *log-odds*, are presented in Equation 2.

(2) $$\;\begin{array}{l} V_{j}^{M}(i)=\log\frac{e_{\text{Mj}}(x_{i})}{q_{\text{xi}}}+\max\left\{\begin{array}{ccc} V_{j-1}^{M}(i-1) & + & \log(t_{M_{j-1}M_{j}})\\ V_{j-1}^{I}(i-1) & + & \log(t_{I_{j-1}M_{j}})\\ V_{j-1}^{D}(i-1) & + & \log(t_{D_{j-1}M_{j}}) \end{array}\right)\\ V_{j}^{I}(i)=\log\frac{e_{\text{Ij}}(x_{i})}{q_{\text{xi}}}+\max\left\{\begin{array}{ccc} V_{j}^{M}(i-1) & + & \log(t_{M_{j}I_{j}})\\ V_{j}^{I}(i-1) & + & \log(t_{I_{j}I_{j}})\\ V_{j}^{D}(i-1) & + & \log(t_{D_{j}I_{j}}) \end{array}\right)\\ V_{j}^{D}(i)=\max\left\{\begin{array}{ccc} V_{j-1}^{M}(i) & + & \log(t_{M_{j-1}D_{j}})\\ V_{j-1}^{I}(i) & + & \log(t_{I_{j-1}D_{j}})\\ V_{j-1}^{D}(i) & + & \log(t_{D_{j-1}D_{j}}) \end{array}\right) \end{array}$$

The terms *e*_*Ij*_(*x*_*i*_)/*q*_*xi*_, relating the emission probabilities (*e*_*Ij*_(*x*_*i*_)) and the background probability distribution (*q*_*xi*_) of belonging to the standard random model, represent a normalized probability of observing the event *x*_*i*_ at state *Ij*. The remaining variables in these equations represent the following: $V_{j}^{M}(i)$ represents the logarithm of the probability of the most likely path ending at state *M*_*j*_ in column *j*, after processing *i* letters from a given sequence. Likewise, $V_{j}^{I}(i)$ and $V_{j}^{D}(i)$ represent the logarithm of the probability of an insertion and deletion, respectively. *t*_*XY*_ represents the probability of transitioning from one state to another (for example, $t_{M_{2}D_{3}}$ represents the probability of transitioning from *M*_2_ to *D*_3_).

### HMMER

**Figure 4:**
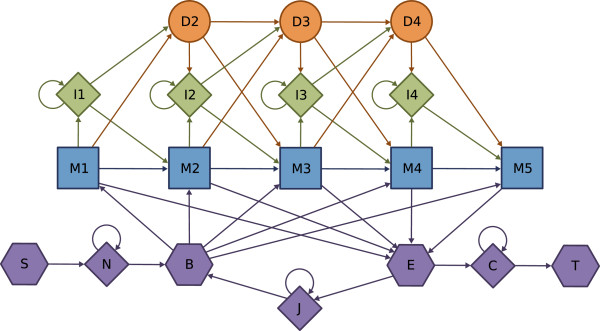
Multihit HMMER3 model.

HMMER [9] is a commonly used software tool that uses HMMs to perform homology search. The original version of HMMER relied on a model architecture entirely similar to Krogh-Haussler’s model. The current version (HMMER 3.1b1 [10]) employs the ‘Plan 7’ model architecture, presented in Figure 4. Although the core of this architecture is still very similar to Krogh-Haussler’s, Plan 7 has no D→I or I→D transitions, which simplifies the algorithm. Furthermore, some special-states are added at the beginning and at the end, in order to allow for arbitrary restarts (thus making it a local alignment) and multiple repeats (multihit alignment). These special states can be parameterized to control the desired form of alignment, such as unihit or multihit, global or local.

This latest HMMER version also introduced a processing pipeline, comprehending a combination of several incremental filters. Each incremental filter is more accurate, restrictive and expensive than the previous one. All of these filters have already been parallelized by Single-Instruction Multiple-Data (SIMD) vectorization using Farrar’s striped processing pattern [3]. The ViterbiFilter, in particular, has been parallelized with 16-bit integer scores. Accordingly, the present work proposes a new parallelization approach of this filter based on Rognes’ processing pattern [4], with a novel strategy to improve the cache efficiency.

## Cache-Oblivious SIMD Viterbi with inter-sequence parallelism

The proposed Cache-Oblivious Parallel SIMD Viterbi (COPS) algorithm represents an optimization of the Viterbi filter implementation in local unihit mode (i.e., the mode corresponding to the original Smith-Waterman local alignment algorithm). Global alignment is not currently supported by the latest version of HMMER.

The presented implementation was developed on top of the HMMER suite, as a standalone tool. A full integration into the HMMER pipeline was deemed unsuitable, since the pipeline was designed to execute a single sequence search at a time, while the proposed approach exploits inter-sequence parallelism, i.e., it concurrently processes several sequences at a time in the SIMD SSE2 vector elements.

A coarse grained structure of the implemented algorithm, when compared with the original HMMER implementation, is presented in Listing 1. The following subsections will describe the several code transformations that were required to implement the proposed processing approach. 

### Rognes’ strategy applied to Viterbi decoding

Although HMMER extensively adopts the Farrar’s intra-sequence vectorization approach, the presented research demonstrates that the inter-sequence parallel alignment strategy that was proposed by Rognes [4] can be equally applied to implement the Viterbi decoding algorithm. The proposed vectorization comprehends the computation of the recursive Viterbi relations, by using three auxiliary arrays to hold the previous values of the Match (M), Insert (I) and Delete (D) states (see Figure 4). After each loop over the normal states, the special states (E and C) are updated. Since the proposed implementation does not support multhit alignments, the J transitions were removed from the original model.

Just like Farrar’s and Rognes’ vectorizations, the implementation that is now proposed uses 128-bit SIMD registers, composed by eight 16-bit integer scores, to simultaneously process eight different sequences. Furthermore, similarly to the HMMER implementation, the scores are discretized by using a simple scaling operation, with an additional bias and saturated arithmetic. Hence, just like the ‘-2.0 nat approximation’ that is used by HMMER, the N → N and C → C transitions were set to zero, and a -2.0 score offset was added at the end. This value approximates the cumulative contribution of N → N and C → C transitions which, for a large *L*, is given by $\log\frac{L}{L+2}$. As a result, the B contributions become constant, since they only depend on the N values (which are constant) and on the J values (which are zero in unihit modes).

**Figure 5:**
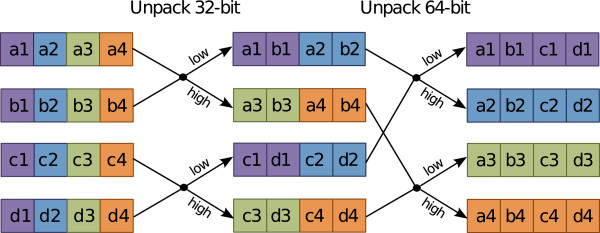
**Emission scores pre-processing using SSE2 unpack instructions.** For illustrative purposes, only 4 sequences (denoted by letters a, b, c and d) were represented. The numeric suffix represents the corresponding index, within the sequence.

A required and important step in this inter-sequence SIMD implementation of the Viterbi decoding is the pre-loading and arrangement of the per-residue emission scores. However, these emission scores depend on the searched sequences and they cannot be predicted, pre-computed and memorized before knowing those sequences. Furthermore, each new batch of 8 sequences to search requires the loading of new emission scores. Rognes’ solution to circumvent this problem can also be adapted to Viterbi decoding and consists on loading the emission scores for the 8 different residues from the 8 sequences under processing (each from its own emission scores array) before starting the main loop of the model (loop A, in Listing 1). To accomplish this, the scores must be transposed from the original *continuous* pattern into a convenient *striped* pattern, by using the unpack and shuffle SSE operations. The implemented processing pattern is illustrated in Figure 5, while the corresponding pseudo-code implementation is presented in Listing 2. 

### Inline pre-processing of the scores

Rognes’ method to pre-load and pre-process the emission scores before each inner loop iteration (i.e., iteration over the model states) suffers from a considerable handicap: it needs an additional re-write of the scores to memory, before the actual Viterbi decoding can start. To circumvent this problem, an alternative approach is herein proposed. Instead of transposing all the emission scores for each tuple of residues in the outer loop of the algorithm (Loop B in Listing 1 (a), over the sequence residues), the transposition was moved inwards to the inner loop (Loop A) and subsequently unrolled for 8 iterations. Hence, each iteration starts by pre-loading 8 emission values: one from each of the 8 continuous arrays. These emission values are then transposed and striped into 8 temporary SSE2 vectors and used in the computation of the next model state for each of the 8 sequences under processing. Hence, the inner loop is unrolled into the 8 state-triplets that are processed by each loop iteration. With this approach, the emission scores can be kept in close memory, thus improving the memory and cache efficiency. Furthermore, the re-writing in memory during this pre-loading phase is also avoided.

To take full advantage of this vectorization approach, the number of considered model states should be always a multiple of 8 (in order to occupy the 8 available SSE channels). Nevertheless, this restriction is easily fulfilled, by padding the model with dummy states up to the next multiple-of-8 state barrier. These dummy states should carry dummy scores (set to -*∞*), so that they have a null influence on the final results, representing a negligible effect on the overall performance. According to the conducted evaluations (further detailed in the latest sections of this manuscript), this optimization of the inlined scores loading procedure leads to an execution time roughly 30% faster than the pre-loading method used by Rognes’ tool.

### Model partitioning

One common problem that is often observed in these algorithms is concerned with the degradation of the cache efficiency when the score arrays exceed the capacity of the innermost-level data caches, leading to an abrupt increase of the number of cache misses and causing a substantial reduction of the overall performance. This type of performance penalties is also present in HMMER Farrar-based ViterbiFilter implementation, whenever larger models are considered.

**Figure 6:**
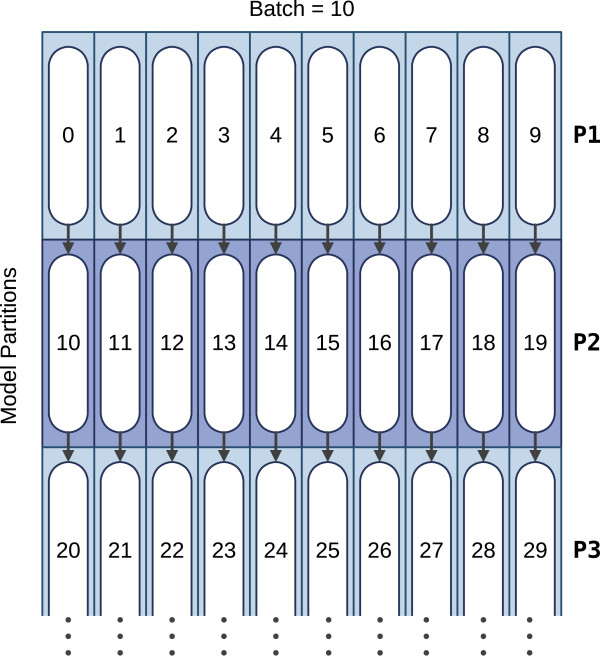
**Processing pattern of the adopted partitioned model, with a batch of length 10.** The numbers represent the processing order of each partition, while the arrows show the inter-partition dependencies.

To circumvent this cache efficiency problem, a *loop-tiling* (a.k.a. *strip-mining*) strategy based on a partitioning of the model states was devised in the proposed implementation, in order to limit the amount of memory required by the core loop. The required code transformations are illustrated in Listing 1(b). Accordingly, the M, I and D model states are split in blocks (or partitions), whose optimal dimension (Maximum Partition (MP) length) is parameterized according to the size and organization of the L1 data (L1D) cache. With this approach, all the defined partitions are now iterated in a new outermost-loop (Loop C, in Listing 1(b)). As a result, the inner loop (Loop A) is substantially shorter and it is now possible to obtain an optimal cache use in loops A and B — the middle loop (Loop B) iterates over the 8 database sequences, while the inner loop (Loop A) iterates over a single partition of model states.The middle loop (Loop B), over the database sequences, mostly re-uses the same memory locations (except for the emission scores) that are accessed in the inner core loop (Loop A). Consequently, these locations tend to be kept in close cache. By limiting this model states loop to a pre-defined number of state-triplets defined by the MP length, it can be assured that the whole sequence loop (Loop B) is kept in cache. Hence, with this optimization, the memory required by the inner loop (Loop A) is always cached in close memory and repeatedly accessed over the whole sequence loop, thereby drastically reducing the occurrence of cache misses. To attain the maximum performance, the MP length should be adjusted in order to achieve an optimal cache occupation, i.e., one that fills the available capacity of the innermost data cache (L1D). The processing pattern resulting from the proposed partitioned model is represented in Figure 6.

Listing 3 presents the pseudo-code of the whole algorithm implementation. The pseudo-code corresponding to the procedures EMISSION_SCORES_PREPROCESS and COMPUTE_STATE_TRIPLET, used in the inner loop (Loop A), are depicted in Listings 2 and 4, respectively. The notation adopted in this pseudo-code is closer to the provided software implementation than equations 1 and 2, defining the algorithm. Accordingly, the variable names re properly adapted. In particular, the *j* indexes were omitted and use *cv* (current value). Likewise, *pv* (previous value) was used to represent the index *j*-1. Hence, variable *Mpv* represents $V_{j-1}^{M}(i)$. Similarly, *Dpv* represents $V_{j-1}^{D}(i)$ and *Ipv* represents $V_{j-1}^{I}(i)$. It is also worth noting that these variables are not arrays. Instead, once the values are computed they are copied to the arrays *Mmx*(*j*), *Dmx*(*j*) and *Imx*(*j*), respectively. The transition probabilities *t*, are stored in 8 arrays (for example *t*_*MI*_, for transitions from match to insert states). The computation of each Match value is split between iterations. Hence, an additional variable *Mnext* is required to carry the partial computed value onto the next iteration. 

Table 1 represents the memory footprint required by the proposed COPS implementation, when compared with the original HMMER ViterbyFilter. *M* represents the model length. At this respect, it is important to note that although the presented approach exceeds the innermost cache capacity sooner, since 8 times more transition scores and 8-fold larger dynamic programming arrays are required in the inner loop, the cumulative amount of cache misses along the time is substantially lower, as a result of the proposed partitioning.

**Table 1:** Memory footprint (in Bytes) required by the proposed COPS implementation, when compared with the original HMMER ViterbyFilter

**Data structure**	**COPS (proposed)**	**ViterbiFilter**
		**(HMMER)**
Mmx, Dmx, Imx	3×*M*×16	3×*M*×2
Transition scores	8×*M*×16	8×*M*×2
Emission match (E.M.) scores	*M*×16	*M*×2
Auxiliary emission array	24×16	–
∼20 aux. variables	20×16	20×16
Total	192×*M*+700	24×*M*+320
Total minus E.M. scores	176×*M*+700	22×*M*+320
Max. *M* to fill a 32 KB cache	$\frac{32768-700}{192}\approx 167$	$\frac{32768-320}{24}\approx 1350$

*M* represents the model length and all the computed scores are represented with 16-bit integers.

Overall, the partitioned COPS implementation has an expected memory footprint of around 240×*M*+900 bytes (corresponding to the original memory requirements of the non-partitioned COPS, plus the additional arrays that are required to store the inter-partition dependencies). It can thereby be estimated an optimal MP value as the maximum model length (*M*) that limits the memory footprint within the size of the L1D cache. Hence the MP length can be determined by:

(3) $$\text{MP}=\frac{\text{size}(L1D)-900}{240}$$

Nevertheless, a conservative tolerance should be considered when approaching this maximum estimate, justified by the sharing of the L1D cache with other variables not correlated with this processing loop, process or thread. In fact, the conducted experimental procedures demonstrated that the actual MP values are very close to the best values that were theoretically estimated: 

• 112 to 120 states, for 32 KB L1D CPUs (e.g. Intel Core, Core2, Nehalem, Sandy Bridge, Ivy Bridge and Haswell);

• around 48 states, for 16 KB L1D CPUs (e.g. AMD Opteron Bulldozer and Piledriver);

•216 to 224 states, for 64 KB L1D CPUs (e.g. AMD Opteron K8, K10, Phenom I and II).

There are, however, two memory blocks that cannot be *strip-mined*: 

• Emission scores, which must be refreshed (re-computed) for each new round of sequence tokens. These values are accessed only once, so it is counter-productive to consider their cacheability.

• Dependencies that must be exchanged between adjacent partitions. The last Match (M), Insert (I) and Delete (D) contributions from each partition have to be carried on in the next partition, and so they have to be saved at the end of each partition. Hence, each partition receives as input one line of previous states, with one state-triplet for each 8-fold round of sequences, and produces as output another line of values to be used by the next partition. These dependencies can be minimized to 3 values per sequence round (*xmxE*, *Mnext* and *Dcv*) after re-factoring the core code and moving the computation of *Mnext* with the 3 state dependencies to the end. The re-factored inner loop code can be seen in Listing 4.

## Methods

To conduct a comparative and comprehensive evaluation of the proposed approach, the COPS algorithm was ran against the ViterbiFilter implementation of HMMER 3.1b1, based on Farrar’s striped vectorization. For such purpose, a benchmark dataset comprehending both DNA and protein data was adopted, covering a wide spectrum of model lengths, ranging from 50 to 3000 model states, with a step of about 100.

In particular, the DNA data consisted on HMMs sampled from Dfam 1.2 database of Human DNA HMMs [11], and the human genome retrieved from the NCBI archive.

As of March 2013, Dfam uses HMMER3.1b1 to create the models. The complete list of HMMs is the following (the length is prefixed to the model name): M0063-U7, M0804-LTR1E, M1597-Tigger6b, M2500-L1M4c_5end, M0101-HY3, M0900-MER4D1, M1683-FordPrefect, M2596-L1P4a_5end, M0200-MER107, M1000-L1MEg2_5end, M1795-L1MB4_5end, M2706-Charlie3, M0301-Eulor9A, M1106-L1MD2_3end, M1961-Charlie4, M2789-L1MC4_3end, M0401-MER121, M1204-Charlie17b, M2101-L1MEg_5end, M2904-L1M2_5end, M0500-LTR72B, M1302-HSMAR2, M2204-CR1_Mam, M2991-HAL1M8, M0600-MER4A1, M1409-MLT1H-int, M2275-L1P2_5end, M0700-MER77B, M1509-LTR104_Mam, M2406-Tigger5.

The protein data consisted on a mix of 13 small and medium-sized HMMs from Pfam 27.0 [12] and 17 large HMMs created with *hmmerbuild* tool from Protein Isoforms sampled from Uniprot, and the NRDB90 [13] non-redundant protein database. The short protein models, from Pfam, were the following: M0063-ACT_5, M0400-Alginate_exp, M0800-Patched, M1201-DUF3584, M0101-Bactofilin, M0500-Lant_dehyd_C, M0900-PolC_DP2, M1301-Orbi_VP1, M0201-Adeno_52K, M0600-Mpp10, M1002-SrfB, M0300-Aldose_epim, M0700-Pox_VERT_large, M1098-CobN-Mg_che.

The longer models used were generated from the following Uniprot Isoforms: M1400-Q8CGB6, M1800-Q9BYP7, M2203-P27732, M2602-O75369, M1500-Q9V4C8, M1901-Q64487, M2295-Q3UHQ6, M2703-Q8BTI8, M1600-Q6NZJ6, M2000-Q9NY46, M2403-Q9UGM3, M2802-Q9DER5, M1700-Q3UH06, M2099-Q8NF50, M2505-O00555, M2898-Q868Z9, M3003-A2AWL7.

The benchmarks were run on two different machines: 

• Intel Core i7 3770 K, with an Ivy Bridge architecture, running at 3.50 GHz with a 32 KB L1D cache;

• AMD Opteron 6276, with a Bulldozer architecture, running at 2.3 GHz with a 16 KB L1D cache.

All the timings were measured as total walltime, by using the Linux ftime function.

## Results and discussion

### Cache misses

To evaluate the cache usage efficiency of the considered algorithms, the number of L1D cache misses for the COPS tool and for the HMMER ViterbiFilter implementations were measured with PAPI performance instrumentation framework [14]. To ensure a broader and more comprehensive coverage of measures, a wider and random dataset of DNA models was considered in this specific evaluation.

**Figure 7:**
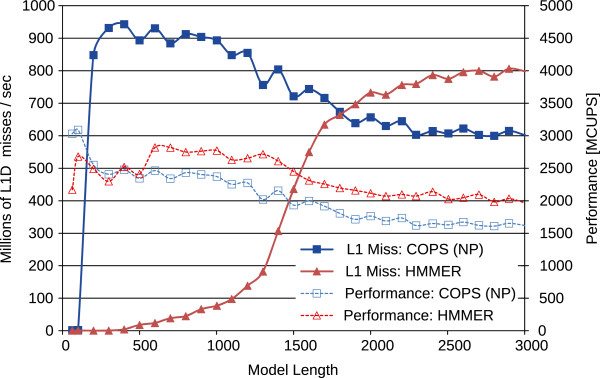
**Cache usage results of HMMER ViterbiFilter and of a *Non-Partitioned* COPS (NP) implementation on the Intel Core i7 with 32 KB of L1D cache.**

When Intel processors (with 32 KB L1D caches) are considered, the theoretical estimates suggested a *critical point* for optimal L1D cache utilization corresponding to models of size *M*≈1350 for the HMMER ViterbiFilter and *M*≈167 for COPS. To confirm the formulated estimation, the experimental procedure started by considering a *non-partitioned* implementation, which was evaluated in conjunction with the corresponding HMMER implementation. The obtained values, illustrated in Figure 7, demonstrate that the theoretically estimated *critical points* coincide very closely with the observed abrupt increases of the L1D cache misses, as well as with the corresponding performance drops, which are strongly correlated in the observed results.

**Figure 8:**
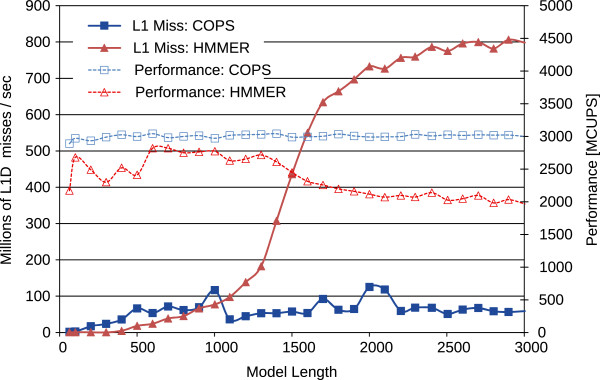
Cache usage results of HMMER ViterbiFilter and of the new partitioned COPS implementation on the Intel Core i7 with 32 KB of L1D cache.

After partitioning, the overall performance of the proposed COPS algorithm behaved remarkably close to what had been predicted, maintaining the same level of caches misses and computation performance for any model length (see Figure 8). As it can be seen in this figure, COPS even managed to be slightly faster than HMMER ViterbiFilter for models up to *M*≈1200 in 32 KB L1D cache machines. For longer models, COPS gains are close to 1.5-fold speedup over HMMER ViterbiFilter, due to the cache degradation observed in HMMER. When compared with the non-partitioned COPS implementation (see Figure 7), the partitioned version was about 50% faster for long models (>1000 bps), demonstrating the remarkable benefits of the proposed partitioned processing approach.

### Performance

**Figure 9:**
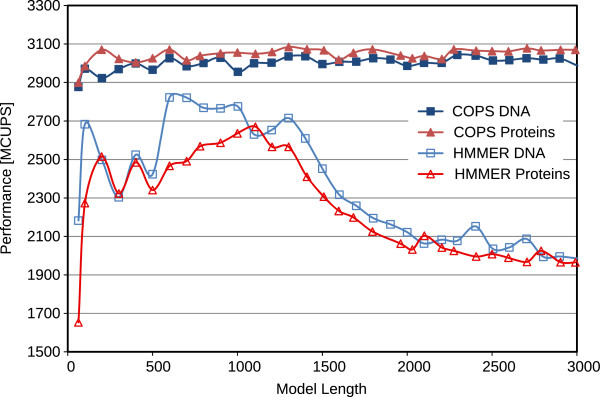
Comparative performance results of the proposed COPS implementation and of HMMER ViterbiFilter, obtained on the Intel Core i7 (32 KB of L1D cache).

**Figure 10:**
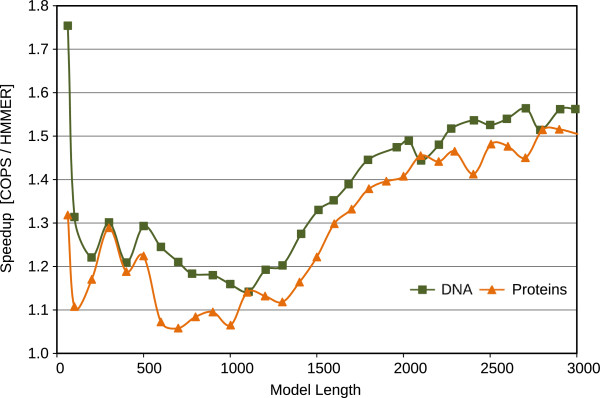
Resulting speedup of the proposed COPS implementation over HMMER ViterbiFilter, obtained on the Intel Core i7 (32 KB of L1D cache).

**Figure 11:**
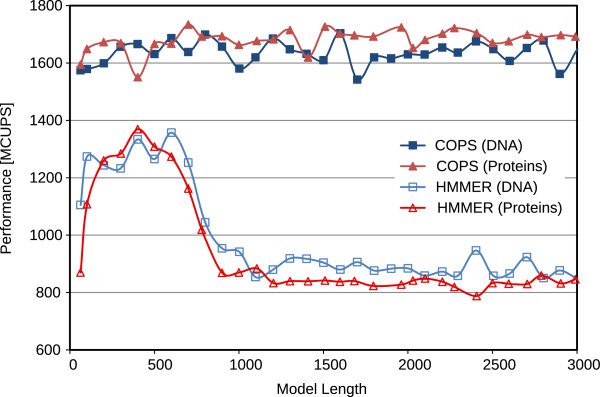
Comparative performance results of the proposed COPS implementation and of HMMER ViterbiFilter, obtained on an AMD Opteron Bulldozer (16 KB of L1D cache).

**Figure 12:**
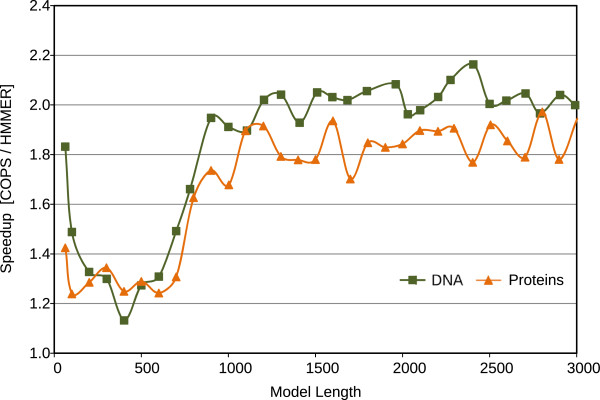
Resulting speedup of the proposed COPS implementation over HMMER ViterbiFilter, obtained on an AMD Opteron Bulldozer (16 KB of L1D cache).

Figures 9 and 10 represent the performance (in Millions of Cell Updates Per Second (MCUPS)) of the two implementations and the observed speedup of the presented COPS approach, when using the Intel Core i7 processor. Figures 11 and 12 represent similar results, observed in the AMD processor.

For short models (< 100 bps), the penalizing overhead of Farrar’s *Lazy-F loop* is clearly evident. As a result, the HMMER ViterbiFilter has a very poor performance on these models. In contrast, the proposed COPS solution does not suffer from this problem and presents a much smaller performance penalty in these small models (mainly from the initialization costs between each inner-loop execution). As a result, with these short models, COPS achieved a considerable 1.7-fold speedup, when compared with HMMER.

For medium-length models (between 100 and 500 bps on 16 KB-L1D machines, and up to ≈1000 bps on 32 KB-L1D machines), the proposed COPS implementation is about as good as HMMER, reducing the observed speedup to about 1.2-fold. These performance values correspond to model lengths where the striped version does not exceed the size of the innermost data cache.

For longer models, from 500 bps or 1000 bps (depending on the L1D size), it can be observed that the performance of HMMER quickly deteriorates as the length of the model increases and the memory requirements of HMMER Farrar-based approach reach the maximum that the innermost L1D caches can provide (usually, 32 KB on Intel and 16 KB on AMD CPUs). In contrast, the proposed inter-sequence COPS is able to consistently maintain the same performance level with increasingly long models, thus achieving a 2-fold speedup on AMD and a 1.5-fold on Intel, against the HMMER version for longer models.

## Conclusions

The main insight of the presented approach is based on the observation that current parallel HMM implementations may suffer severe cache penalties when processing long models. To circumvent this limitation, a new vectorization of the Viterbi decoding algorithm is proposed to process arbitrarily sized HMM models. The presented algorithm is based on a SSE2 inter-sequence parallelization approach, similar to the DNA alignment algorithm proposed by Rognes [4]. Besides the adopted alternative vectorization approach, the proposed algorithm introduces a new partitioning of the Markov model that allows a significantly more efficient exploitation of the cache locality. Such optimization, together with an improved loading of the emission scores, allows the achievement of a constant processing throughput, regardless of the innermost-cache size and of the dimension of the considered model.

In what concerns the partitioning, the proposed implementation was based on the observation that the poor cache performance of HMMER is related to the size of the model and to the fact that it is necessary to update all the states in the model for every letter of a query sequence. As a result, large models will force recently computed values out of cache. When this phenomena occurs for every letter in a query, it naturally results in a significant bottleneck.

We speculate that a similar phenomena occurs for the striped pattern of Farrar, in which case our partitioning technique could prove useful. Still, Farrar’s algorithm processes one single query at a time, instead of 8. Therefore, the slowdown should only occurs for models 8 times larger, i.e., models of size larger than 10800.

According to the extensive set of assessments and evaluations that were conducted, the proposed vectorized optimization of the Viterbi decoding algorithm proved to be a rather competitive alternative implementation, when compared with the state of the art HMMER3 decoder. Being always faster than the already highly optimized HMMER ViterbiFilter implementation, the proposed implementation provides a constant throughput and proved to offer a processing speedup as high as 2, depending on the considered HMM model size and L1D cache size.

Future work may also extend this approach to Intel’s recent instruction-set extension AVX2, allowing the processing of twice more vector elements at a time.

## Availability and requirements

**Project name:** COPS (Cache-Oblivious SIMD Viterbi with Inter-Sequence Parallelism)**Project home page:**https://kdbio.inesc-id.pt/~lsr/COPS**Operating system(s):** Linux**Platform independent Programming language:** C**requirements:** gcc, make**License:** a variation of the Internet Systems Consortium (ISC) license.**Restrictions to use by non-academics:** Referencing this work.

## Competing interests

The authors declare that they have no competing interests.

## Authors’ contributions

MF analyzed the problem and implemented the prototype, which was subsequently used for profiling and evaluation. NR and LR introduced the problem, along with an initial analysis, and recommended experimental approaches. All authors read and approved the final manuscript.
